# Mineral Bone Disorders in Kidney Disease Patients: The Ever-Current Topic

**DOI:** 10.3390/ijms232012223

**Published:** 2022-10-13

**Authors:** Lilio Hu, Angelodaniele Napoletano, Michele Provenzano, Carlo Garofalo, Claudia Bini, Giorgia Comai, Gaetano La Manna

**Affiliations:** 1 Nephrology, Dialysis and Renal Transplant Unit, IRCCS—Azienda Ospedaliero-Universitaria di Bologna, Alma Mater Studiorum University of Bologna, 40126 Bologna, Italy; 2 Renal Unit, University of Campania “L. Vanvitelli’’, 80138 Naples, Italy

**Keywords:** CKD–MBD, kidney, epidemiology, prognosis, cardiovascular, ESKD

## Abstract

Chronic kidney disease (CKD) is a complex and multifactorial disease, and one of the most prevalent worldwide. Chronic kidney disease–mineral bone disorders (CKD–MBD) with biochemical and hormonal alterations are part of the complications associated with the progression of CKD. Pathophysiology of CKD–MBD focused on abnormalities in serum levels of several biomarkers (such as FGF-23, klotho, phosphate, calcium, vitamin D, and PTH) which are discussed in this review. We therefore examine the prognostic association between CKD–MBD and the increased risk for cardiovascular events, mortality, and CKD progression to end-stage kidney disease (ESKD). Lastly, we present specific treatments acting on CKD to prevent and treat the complications associated with secondary hyperparathyroidism (SHPT): control of hyperphosphatemia (with dietary restriction, intestinal phosphate binders, and adequate dialysis), the use of calcimimetic agents, vitamin D, and analogues, and the use of bisphosphonates or denosumab in patients with osteoporosis.

## 1. Introduction

Chronic kidney disease (CKD) has emerged as one of the most prevalent noncommunicable disease, affecting about 10% of the general population worldwide [1]. The glomerular filtration rate (GFR) decline, which is a characteristic of the course of disease, is associated with the onset of severe complications such as arterial hypertension, anemia, hyperkalemia, metabolic acidosis, and mineral bone disorders. Among them, mineral bone disorders represent a clinical condition, which, when present and not sufficiently controlled, forecast a very high risk of death, cardiovascular (CV) events, and CKD progression to end-stage kidney disease (ESKD). Slowing kidney disease progression is a very important aim of clinical research given the poor prognosis of patients with advanced CKD and ESKD.

Mineral bone disorders (also called with the acronyms of ‘chronic kidney disease—mineral and bone disorder’, CKD–MBD) are characterized by biochemical and hormonal alterations with a significant increased risk for bone fractures, CV events, mortality, and CKD progression [2]. The main alterations of CKD–MBD encompass hyperphosphatemia, hypocalcemia, low serum levels of vitamin D, and an increased secretion of parathyroid hormone (PTH) from the parathyroid glands (secondary hyperparathyroidism, SHPT). All these conditions trigger deep alterations of bone and mineral metabolism, renal osteodystrophy, and extra-skeletal calcification, which, taken together, forecast a poor prognosis. Owing to this background, several strategies have been adopted at improving the management of CKD patients. Overall, the main therapeutic tools include the reduction in serum phosphorus levels through diet or phosphate binders; nutritional vitamin D or VDRA (vitamin D receptors activators); and the use of calcium mimetics. The aim of the therapy is to maintain phosphorus levels, calcium, and PTH within ‘ideal’ target values in order to preserve bone health and decrease soft tissue and vascular calcifications. Another important therapeutic strategy is represented by the surgical intervention with parathyroidectomy that should be considered in cases nonresponsive to medical therapy [3]. Although many therapeutic step forwards have been accomplished in the last 20 years, the management of CKD–MBD is a challenging topic and residual risk associated with CKD–MBD is very high. The aim of the present review is to summarize pathophysiology of CKD–MBD disorders in CKD patients, prognostic aspects of disease, and the novel therapeutic strategies especially in the perspective of a personalized management.

## 2. Pathophysiology of CKD–MBD

**Figure 1:**
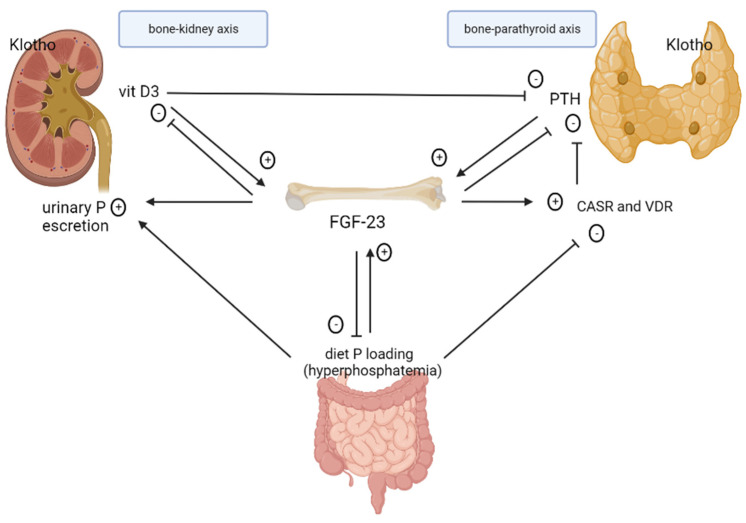
The interplay among FGF-23, PTH, vitamin D3, and phosphorus in CKD. Secretion of FGF-23 is stimulated by increased levels of PTH, 1,25(OH)D3 and phosphorus from diet loading. On the other side, FGF-23 inhibits PTH secretion, decreases levels of 1,25(OH)D3, reduces intestinal absorption of alimentar phosphorus, inhibits phosphorus reabsorption in the proximal tubule leading to increased urinary excretion. Furthermore, 1,25(OH)D3 suppresses PTH, and hyperphosphatemia reduces CASR sensibility, directly affecting PTH synthesis.

According to the Kidney Disease: Improving Global Outcomes (KDIGO) 2006 guidelines, CKD–MBD is defined as ‘‘a systemic disorder of mineral and bone metabolism due to CKD manifested by either one or a combination of the following: (a) abnormalities of calcium, phosphorus, PTH, or vitamin D metabolism; (b) abnormalities in bone turn-over, mineralization, volume, linear growth, or strength; or (c) vascular or other soft tissue calcification’’ [4]. Hence, CKD–MBD involves a number of interconnections between the kidneys, parathyroid glands, bone, and intestine (Figure 1).

Although CKD–MBD generally starts early in the course of the CKD, it becomes apparent with secondary hyperparathyroidism, hyperphosphatemia, and hypocalcemia only when GFR falls below 45–50 mL/min/1.73 m^2^ (based on the different methods to assess GFR) and it worsens with its progression (stage 3b–5) [5]. Since Albright’s first studies on the close relationship between calcium, phosphate, and parathyroid hormone (PTH) [6], our understanding of mineral metabolism has expanded considerably and the complex mechanisms behind CKD–MBD have been progressively disclosed. However, a lot of unmet needs are still present. In the 1960 original exposition of the “intact nephron hypothesis” [7], Briker et al. stated that although kidney disease consists of a diminished number of nephrons, the remaining nephrons undergo adaptive changes and increase their function to compensate for the damaged nephrons and maintain homeostasis of any given solute. These adaptations consist of an increase in single nephron GFR, decreased tubular reabsorption, and increased tubular secretion, but they can actually carry some consequences on other systems and result in abnormalities of the uremic state (the “trade-off theory”) [8,9]. This pathophysiologic theory affects CKD–MBD as well. In fact, during the course of CKD, the progressive decrease in GFR leads to phosphate retention; however, it has been demonstrated that serum phosphate level may remain normal until the advances stages of CKD [10] due to a compensatory increase in PTH and fibroblast growth factor 23 (FGF-23), two pivotal main hormones involved in the regulation of renal phosphate handling [11]. 

The identification of FGF-23 and its coreceptor Klotho has resulted in a new perspective of the “trade-off” theory. FGF-23 has both short-term beneficial (reduction in serum phosphate level) and long-term adverse effects, as its circulating concentration progressively increases while GFR falls down, achieving levels that are several hundred times the normal range in late CKD stages [12]. Thus, FGF-23 is likely involved in the development of SHPT.

### 2.1. Role of FGF-23 in CKD

FGF-23 is primarily synthesized by osteocytes and osteoblasts in response to oral phosphate loading and increased serum levels of 1,25-dihydroxyvitamin D3 (1,25(OH)2D3) and PTH. Additional factors that regulate FGF-23 production and secretion include ionized calcium, reduced levels of iron deposits, the RAAS, oxidative stress, and inflammation [13]. The physiologic action of FGF-23 on target organs is mediated by FGF-23 receptors (FGFRs) that require the presence of the coreceptor αKlotho which increases FGF-23 affinity to FGFR [14]. In the kidneys, FGF-23 reduces phosphate reabsorption and promotes urinary excretion of phosphate by down-regulation of the expression of luminal type 2 sodium-dependent phosphate cotransporters NaPi2a and NaPi2c in the proximal tubule [15]; in the distal tubule, FGF-23 increases calcium and sodium reabsorption by rising apical expression of the epithelial calcium channel TRPV5 and the sodium-chloride cotransporter [16]. In early CKD, FGF-23 plays a protective role as it triggers adaptive changes to restore normophosphatemia; therefore, it is a biomarker of abnormal phosphate homeostasis. Isakova et al. demonstrated that FGF-23 increases earlier than serum PTH [12]. In a prospective study of patients with mild-to-moderate CKD, higher serum FGF-23 levels were shown to predict a more rapid progression toward ESKD [17]. FGF23 secreted from bone mainly acts on the FGFR–Klotho complex expressed in the kidney (the bone−kidney axis) and parathyroid gland (the bone−parathyroid axis) [14]. FGF-23 also suppresses synthesis and promotes degradation of 1,25(OH)2D3 by inhibition of the 1-α-hydroxylase (CYP27B1) and by stimulation of 24-hydroxylase (CYP24A1) [18], respectively. Therefore, FGF-23 functions as a counterregulatory hormone for vitamin D [19]. Furthermore, FGF-23 leads to the activation of the renin–angiotensin–aldosterone system (RAAS) through the suppression of the expression of the angiotensin-converting enzyme-2 (ACE-2) in the kidney that contrasts the activity of the angiotensin-converting enzyme (ACE) [20]. FGF-23 also increases indirectly renin expression through reduction in 1,25(OH)2D3 [21]. In the parathyroid glands, FGF-23 inhibits production and secretion of PTH, but also increases parathyroid calcium-sensing receptor (CaSR) and vitamin D receptor (VDR) expression, further contributing to the suppression of PTH by this hormone [22]. In the intestine, FGF-23 reduces intestinal absorption of alimentary phosphorus through inhibition of NaPi2b cotransporter activity [23] and reduction in 1,25(OH)2D3 serum concentration [18], inducing a negative phosphate balance. As a result, in patients with CKD, high levels of FGF-23 attenuate hyperphosphatemia, decrease levels of active vitamin D, and inhibit PTH synthesis and secretion.

After a successful kidney transplant with stable graft function, FGF-23 levels decrease significantly within normal limits [24].

### 2.2. Role of Klotho in CKD

Klotho is a type I membrane β-glycosidase-like protein that confers tissue specificity to FGF-23 [13] and its expression is positively regulated by 1,25(OH)2D3 [25]. Klotho deficiency in experimental mice models leads to a phenotype characterized by an altered calcium/phosphate metabolism with hyperphosphatemia, secondary hyperparathyroidism, vascular calcification, cardiac hypertrophy, premature aging, and a shortened lifespan [25,26]. During CKD, the reduction in plasma Klotho levels causes disorder of FGF-23/Klotho axis. A low plasma level of vitamin D in CKD patients reduces Klotho expression in the kidney and in the parathyroid glands, making these organs resistant to FGF-23 [27]. A metanalysis conducted over 15 cohort studies including a total of 431 kidney transplant recipients and 108 living kidney donors showed that, compared to serum levels before surgery, Klotho decreased in kidney donors after nephrectomy, whereas it significantly increased in recipients after transplantation, leading to correction of phosphate homeostasis. [28].

### 2.3. Role and Regulation of Phosphate

Phosphate is pivotal for many cellular functions, as a constituent of DNA, cell membrane lipids, high-energy phosphate, second messengers, and protein phosphorylation [29]. Phosphate homeostasis is conserved through three sodium-dependent phosphate co-transporters (NaPi-2a, NaPi-2b, and NaPi-2c) whose expression is regulated by the serum phosphate concentration itself [30] and different hormonal systems. Low serum phosphate levels stimulate NaPi-2b expression in the intestine, enhancing absorption of phosphorus from the diet, and expression of NaPi-2a and NaPi-2c in the kidney proximal tubule, maximizing the reabsorption of filtrated phosphate and minimizing its urinary excretion. The main hormones regulating phosphate balance are FGF-23/klotho and PTH [31]. In early CKD, despite phosphate overload, normophosphatemia is maintained by a compensatory increase in both circulating PTH and FGF-23 concentrations, which downregulate the expression of NaPi-2 cotransporters with phosphaturia effects [15]. 

In advanced CKD, when GFR declines below 30–40 mL/min/1.73 m^2^, there is a dysregulation of the FGF23–Klotho axis: increased FGF-23 levels are no longer able to enhance the excretion of phosphate in the remaining functioning nephrons, eventually leading to the development of overt hyperphosphatemia, which stimulates further secretion of FGF-23 from the bone. At the same time, high levels of FGF-23 suppress 1,25(OH)2D3 production [18], which contributes to promoting secretion of PTH, contributing to the development of a vicious cycle. Hyperphosphatemia promotes hyperparathyroidism through the induction of hypocalcemia, decreased formation of 1,25(OH)2D3, and increased PTH gene expression. The raising of plasma phosphate concentration significantly inhibits CaSR activity via a noncompetitive antagonism [32] and directly affects PTH synthesis by promoting the stability of PTH mRNA [33]. With a progressive worsening of renal function, hyperphosphatemia, and low levels of vitamin D favor hyperparathyroidism, which appears to occur after FGF-23 levels increase to maladaptive concentrations in patients with ESKD [12,34]. In the case-cohort CRIC study, it has been demonstrated that increasing FGF-23 levels are independently associated with increased risk for kidney replacement therapy (KRT) [35]. It has been demonstrated that increased levels of serum phosphate are responsible for a higher risk of CKD progression to ESKD in normoalbuminuric CKD patients, thus testifying a very important role of this marker in revealing residual risk in CKD patients [36].

### 2.4. Role of Calcitriol

Also known as calcitriol, 1α,25-dihydroxyvitamin D3 is the active form of vitamin D synthesized in renal proximal tubules by 1-α-hydroxylase (CYP27B1), which catalyzes the hydroxylation of 25-hydroxyvitamin D (25(OH)D). It is degraded by 24-hydroxylase (CYP24A1) that converts 1,25(OH)2D3 to inactive metabolites [37]. Equilibrium in the reciprocal regulation between 1α-hydroxylase and 24-hydroxylase maintains the equilibrium in calcitriol activity. Synthesis of 1-α-hydroxylase is induced by hypocalcemia and PTH, whereas 24-hydroxylase activity is potentiated by hyperphosphatemia and FGF-23 [18]. Calcitriol activates its cellular receptor (vitamin D receptor or VDR) expressed on target organs, which alters the transcription rates of target genes responsible for the biological responses. It inhibits parathyroid cell proliferation and suppresses PTH gene expression not only directly, but also indirectly, by enhancing the absorption of dietary calcium and phosphate from the intestinal tract. In the bone, it stimulates FGF-23 production from osteocytes [37]. In patients with CKD, the reduction in synthesis or activity of calcitriol by higher levels of FGF-23 and hyperphosphatemia causes hyporesponsiveness of the VDR and a reduced expression of the CaSR on the parathyroid glands, which leads to increased synthesis of PTH and parathyroid glands hyperplasia [38].

### 2.5. Secondary Hyperparathyroidism

SHPT is characterized by increased synthesis and secretion of PTH from parathyroid glands in progressive CKD in response to systemic alteration of mineral homeostasis, such as hyperphosphatemia, hypocalcemia, and reduced 1,25(OH)2D3 [39]. 

In patients with advanced CKD, although FGF-23 binds to the FGFR–Klotho complex in the parathyroid glands to inhibit PTH production, the ability of FGF-23 to suppress PTH is lost due to decreased Klotho [40]. 

**Figure 2:**
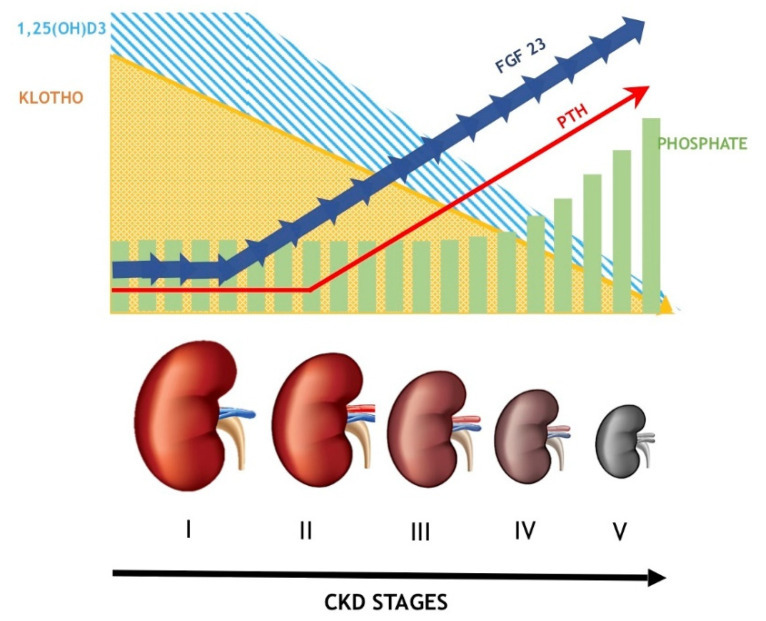
Correlations between serum levels of Klotho, FGF-23, vitamin D, phosphate, and PTH during CKD progression.

The primary regulation of PTH secretion is mediated by calcium ions in the extracellular fluid that directly bind and activate calcium-sensing receptors (CaSR) expressed on parathyroid cells, leading to an increase in intracellular calcium that reduces PTH release [41]. On the contrary, in hypocalcemia conditions, lowered intracellular calcium leads to increased PTH production and secretion. Disturbances of calcium metabolism may be caused by high serum phosphate levels that bind to serum calcium, low oral calcium intakes, impaired intestinal calcium absorption, skeletal resistance to the action of PTH, and reduced CaSR expression in the parathyroid cell. In the kidney, in response to hypocalcemia and hyperphosphatemia, PTH tends to enhance proximal tubule calcium reabsorption and phosphate excretion. In the skeleton, PTH enhances osteoclastic bone resorption that leads to mobilization of calcium and phosphate, mainly from cortical bone [34]. PTH also increases FGF-23 release from mature osteoblasts and osteocytes. In contrast to FGF23, PTH upregulates expression of the 1-α-hydroxylase gene (CYP27B1) and increases 1,25(OH)2D3 production [39], which in turn stimulates intestinal calcium and phosphorus absorption. The enhanced overproduction of PTH in SHPT results in elevated serum calcium and phosphorus levels, which are a consequence of high bone turnover and are linked to the development of vascular calcification of the medial layer of arterial vessels. Medial calcification occurs as a result of both local inflammation and phenotype change in vascular smooth muscle cells to osteoblast-like cells [42]. This phenotype switch is initiated by hyperphosphatemia, hypercalcemia [43], and oxidative stress [44]. Moreover, hyperphosphatemia and hypercalcemia stimulate the release of vascular smooth muscle cell-derived matrix vesicles with the deposition of hydroxyapatite [45]. Figure 2 shows FGF-23, Klotho, phosphate, PTH, and 1,25(OH)D3 levels at different stages of CKD.

### 2.6. Renal Osteodystrophy

Renal osteodystrophy is a component of the mineral and bone disorders related to CKD (CKD–MBD) and it is the term used to describe the spectrum of abnormalities in bone morphology that develops in patients with CKD [4]. Skeletal abnormalities occur in almost the total of stage 5 CKD patients. Aside from the greater risk of bone fracture within this patient group compared to general population [46], bone abnormalities also have significant consequences over mortality and cardiovascular disease [34]. The gold standard for the evaluation and diagnosis of renal osteodystrophy is bone biopsy. An expanded classification based on parameters of bone turnover, mineralization, and volume (TMV system) is recommended to be used to assess bone pathology [4]. The TMV system classifies renal osteodystrophy as: osteitis fibrosa or advanced hyperparathyroid-related bone disease, which is characterized by high bone turnover due to SHPT; adynamic bone disease, which represents the major bone disease in peritoneal and hemodialysis patients and is characterized by low bone turnover due to excessive suppression of PTH with normal mineralization; osteomalacia, which is characterized by low turnover with abnormal mineralization, where the bone volume may be low to medium, depending on the severity and duration of the process, and it is commonly due to aluminum toxicity in bone at a time when aluminum-based phosphate binders were used; and mixed uremic osteodystrophy, which is characterized by abnormal mineralization with either high or low bone turnover [4].

Alterations in bone quality in both high-turnover and low-turnover bone diseases may contribute to the diminished mechanical competence of bone in CKD. Low-turnover bone is manifested by changes in microstructural parameters (cancellous bone volume, and trabecular thickness), whereas bone with high-turnover is manifested by changes in material composition and nanomechanical properties (mineral-to-matrix ratio, carbonate-to-phosphate ratio, hardness, and shape-independent material stiffness) [47]. Because of the high cost and invasiveness of bone biopsy, in most cases several bone biomarkers are used for the assessment of renal bone disease [48]. PTH and bone-specific alkaline phosphatase (b-ALP) are the main markers of bone turnover, being able to discriminate high from nonhigh bone formation rate/bone surface (BFR/BS) rate [49]. Markers of bone formation include b-ALP; osteocalcin, which plays a vital role in osteoid mineralization, and which originates from osteoblasts and odontoblasts; C-terminal and N-terminal propeptide of type I procollagen (PICP, PINP), which arise from proliferating osteoblasts and fibroblasts. Markers of bone resorption include bone sialoprotein, tartrate-resistant acid phosphatase (TRAcP), carboxyterminal crosslinked telopeptide of type I collagen (CTX-I or beta-CrossLaps), amino-terminal crosslinked telopeptide of type I collagen (NTX-I), hydroxylysine glycosides, and hydroxyproline [50].

## 3. Association between CKD–MBD and Prognosis

Patients with CKD are at increased risk of cardiovascular mortality as demonstrated in many studies and this risk increases progressively as renal function declines [51,52].

Cardiovascular mortality in patients affected by CKD is related to traditional risks factors (e.g., age, hypertension, diabetes, dyslipidemia, and smoking habits) and non-traditional risks factors associated with CKD condition, such as chronic inflammation, anemia, and mineral metabolism disorders [53]. CKD–MBD is characterized by abnormalities in the serum levels of several biomarkers such as calcium, phosphorus, PTH, vitamin D, FGF23, and Klotho. This dysregulation can cause a pathological phenomenon characterized by the deposition of calcium-phosphate salts in vascular tissues. Vascular calcification can develop in the intima layer of vessel wall, as frequently occurs in patients with dyslipidemia or in the tunica media of vessels and this is more typical for CKD–MBD. This process was previously considered a passive deposition of salts in blood vessels, cardiac valves, and heart, but recent studies demonstrated that a number of pathways are involved in the pathophysiology of this event. Phenotypic change in vascular smooth muscle cells (VSMC) seems to be an essential step for vessel calcification. In presence of hyperphosphatemia, Pit-1, a type III Na+ dependent phosphate transporter found in many tissues, also on VSMC, induces phosphorylation of Erk 1/2, which in turn leads to osteochondrogenic differentiation of VSMC. This process causes vascular calcification and a consequent increase in wall stiffness, hypertension, left ventricular hypertrophy, hypoperfusion of cardiac tissue, and CV mortality [54,55].

Hyperphosphatemia is a crucial point for the management of CKD patients, especially in dialysis patients, because therapeutic strategies are often ineffective in maintaining phosphorus serum levels within target values. A recent study that collected data from 17,414 HD patients demonstrated that a scarce targeting of phosphate in the first weeks after baseline was strongly associated with CV fatal events. Moreover, a better survival was observed in these patients if phosphorus values were lower than 4.5 mg/dL [56]. The results of a meta-analysis conducted in 2017 by Hou et al., including 9 cohort studies and 1,992,869 HD patients, showed that the highest and also lowest phosphorus levels were associated with an increased risk of all-cause mortality [57]. Moreover, Dhingra et al. demonstrated that also in healthy individuals with normal renal function and without cardiovascular disease, higher serum phosphorus levels are associated with an increased risk of CV events [58]. In patients with already established CKD, hyperphosphatemia is a risk factor for CKD progression to ESKD, even in absence of proteinuria [36]. This can be considered a very important finding when considering that CKD patients without proteinuria are raising in renal clinics and that residual CV and renal risk in these patients is extremely high. 

According to Bellasi et al., CKD patients with phosphate levels ≥4.3 mg/dL have an increased risk of progression to ESKD and mortality [59]. Another study by Sim J.J. and colleagues, which included 94,989 subjects with normal renal function, had the aim to assess if an association exists between higher serum phosphate levels and risk of ESKD; the study population was further divided in phosphorus quartile ranges: 1.9–3.0 mg/dL, 3.1–3.4 mg/dL, 3.5–3.8 mg/dL, and 3.9–5.7 mg/dL. The results showed that higher serum phosphorus levels were associated with greater risk for ESKD and mortality, with an HR 1.48 (95% CI, 0.96–2.28) in the fourth quartile, compared with the first phosphorus quartile [60]. The mechanism by which high phosphorus levels worsen outcomes and renal function are not fully understood. Sahoko et al. studied on transgenic (TG) rats, overexpressing type III Pi transporter Pit-1, the effect of Pi overload on podocytes kidney function in vivo. They found that Pi overload can induce podocyte injury, resulting in the progress of glomerular sclerosis in the kidney [61].

FGF23 is another important molecule involved in CKD–MBD and poor prognosis. FGF 23 actions on target tissues by stimulating FGF receptors (FGFR) and Klotho, which acts as a coreceptor. The myocardium was the first organ that was shown to respond to circulating FGF-23 in a Klotho-independent way [62]. FGF-23 effect on cardiac myocytes is mediated through FGFR 4 which causes activation of PLCg/calcineurin/NFAT signaling; this pathway is well known to be an inducer of left ventricle hypertrophy (LVH) [63]. A recent study conducted on cultured human atrial fibroblasts by Lee et al. showed that FGF-23 may activate FGF receptor 1, enhancing human atrial fibroblast activity [64]. A recent meta-analysis of seven observational studies (1406 ESKD patients) conducted by Yang et al. showed that elevated serum FGF-23 is a marker of poor prognosis in ESKD patients, and is associated with increased risk of all-cause mortality [65]. Another study by Isakova T. et al. showed the same results in a population of 3879 patients with CKD 2–4 and even in these patients with earlier stages of CKD, FGF-23 is a risk factor for mortality, where participants with a baseline estimated GFR > 30 mL/min/1.73 m^2^ were associated with greater risk of ESKD [66]. In a large study of 13,448 participants, where mean GFR was 97 mL/min per 1.73 m^2^, Rebholz et al. demonstrated that higher baseline serum levels of FGF-23 are positively correlated with a higher risk of incident kidney disease [67].

Other studies relating to PTH have shown a “U”-shaped association between PTH values and increased risk of mortality. Similarly, higher PTH levels forecast CKD progression and the increase in PTH over time is also associated with CKD progression, confirming the importance of repeated measures over this biomarker to better stratify the individual prognosis in CKD patients [68]. 

According to KDIGO 2017, in patients with CKD G5D, are suggested PTH levels in the range of 2 to 9 times the upper normal limit [69]. In fact, both groups of HD patients with reduced and increased PTH levels in either direction out of this range are associated with poor prognosis. Floege J. et al. demonstrated that in patients on chronic hemodialysis treatment, PTH levels < 75 pg/mL or >600 pg/mL are associated with an increased risk of death (HR 2.10 and HR 1.46, respectively) compared to HD patients who were within target range [70].

For all these reasons, in the complex scenario of CKD–MBD, management of metabolic alterations correlated to hyperparathyroidism is crucial to minimize the adverse consequences such as CV risk and CKD progression associated with this condition. A detailed summary of the observational studies in MBD is reported in Table 1.

## 4. Risk Reduction Induced by Specific Treatments Acting on CKD–MBD

The management of CKD–MBD is largely based on several strategies to prevent the adverse complications associated with SHPT. The current KDIGO guideline recommends treatment of SHPT based on repeated measures of the biochemical markers of disordered mineral bone metabolism, which include serum phosphate, calcium, parathyroid hormone, and 25-hydroxyvitamin D. PTH target in patients on dialysis is set on two to nine times the normal range, according to KDIGO [71], and 150–300 pg/mL according to KDOQI [72]. In case of overt hyperphosphatemia, guidelines suggest lowering phosphate levels toward the normal range (3.5–5.5 mg/dL) because there is an absence of data supporting that efforts to maintain phosphate in the normal range are of benefit [69]. A detailed summary of the randomized clinical studies that compare different treatments acting on CKD–MBD is reported in Table 2.

### 4.1. Management of Hyperphosphatemia

In patients with CKD G3a-5, updated guidelines recommend that decisions about phosphate-lowering treatments should be based on progressive or persistent hyperphosphatemia. Early prevention of hyperphosphatemia is not currently supported by data derived from randomized studies, even if observational evidence suggests to maintain serum phosphorus levels as low as possible [36]. Phosphate-lowering treatments include diet, dialysis, and phosphate-binding agents.

#### 4.1.1. Dietary Restriction of Phosphate

The daily phosphate intake of a healthy person is approximately 1000–2000 mg. In adult patients with CKD undergoing dialysis with serum phosphate concentrations of 4–4.5 mg/dL, the daily dietary phosphate intake should be limited to approximately 800 mg [73]. The dietary phosphate content is generally proportional to protein intake on the basis of the following equation by Kalantar–Zadeh et al.: Dietary P (mg) = 78 + 11.8 × protein intake (grams) [74]. 

Therefore, a strict limitation on the dietary phosphate intake can, however, lead to protein deficiency and malnutrition due to reduced protein intake. To reach the goal of dietary phosphate intake restriction while maintaining the high protein intake required in dialysis patients, it is recommended that patients select foods with a low phosphorus to protein (P–P) ratio, such as egg white or protein from vegetable-based sources, and prefer cooking procedures that induce food demineralization, such as boiling [75]. Foods with high P–P ratio, such as egg yolk, and those with high amounts of phosphorus-containing preservatives such as certain carbonated beverages and processed cheese and meat should be avoided. A higher dietary phosphate intake and a higher dietary P–P ratio has been reported to be associated with an increased mortality [76]. Moreover, gastrointestinal absorption rate of phosphorus is higher for inorganic phosphate (40–60%) and natural phosphate derived from animal-based food (80–100%), as compared with vegetable sources (20–40%) [75].

#### 4.1.2. Intestinal Phosphate Binders

Phosphate binders sequester dietary phosphate within the gastrointestinal tract forming a non-absorbable complex with phosphate, so they prevent its absorption and enhance its fecal excretion. The binding ability is limited to approximately 200–300 mg of phosphate daily, thus a controlled dietary phosphate load is still required. These drugs are generally well tolerated and can be taken during or after meals. The main classes of phosphate-lowering agents are calcium-based phosphate-binders (CBP), non-calcium-based phosphate-binders (NCBP), and aluminum-based phosphate-binders. 

The use of aluminum containing phosphate binders is nowadays avoided due to the risk of development of aluminum-related toxicity, including osteomalacia and encephalopathy [77].

Calcium-containing phosphate binders (calcium acetate and calcium carbonate) have a slightly lower phosphate-binding capacity than aluminum-based phosphate binders but have no risk of aluminum accumulation and they are more cost-effective. However, they may cause positive calcium balance and elevated calcium-phosphorus (Ca × P) product level, which is linked to hypercalcemia, and cardiac and vascular calcification [78]. Block et al. have found that coronary artery calcification progressed more quickly, and mortality rate was higher in patients taking CBP compared with those receiving NCBP (HR: 3.10, 95% CI 1.23–7.61) [79]. Moreover, the intense use of CBP has been associated with suppression of intact PTH, which leads to the development of low bone turnover and bone loss over time [80].

Calcium acetate/magnesium carbonate (CaMg) is a combination phosphate binder, and it is an effective alternative to pure calcium salts to reduce calcium exposure, combined with the potential benefits associated with increased serum magnesium levels, such as reduced cardiovascular calcifications, hypertension, and mortality risk [81]. In the CALMAG (calcium acetate magnesium carbonate evaluation) study, a randomized multicenter clinical trial enrolling 326 patients, authors found that CaMg was noninferior compared with sevelamer hydrochloride in controlling serum phosphorus levels. Moreover, a minimal increase in total serum calcium and a small increase in serum magnesium have been observed [82].

Based on this evidence, KGIDO guidelines suggest restricting the dose of calcium-based phosphate binders [71]. The results of the meta-analysis conducted by Jamal et al., involving 4622 patients from 11 randomized trials, showed that NCBP are associated with a decreased risk of all-cause mortality compared to CBP (risk ratio 0.78, 95% CI 0.61–0.98) [83]. Another meta-analysis of 18 randomized controlled trials performed in 3676 dialysis patients demonstrated a significantly lower incidence of coronary artery calcification and a significant higher bone formatting rate in NCBP compared with CBP treatment, with equal efficacy on serum phosphate control [84].

The main calcium-free and aluminum-free phosphate-lowering agents are sevelamer and lanthanum carbonate. These drugs are much more used since they do not cause calcium overload and hypercalcemia. Sevelamer is a weakly basic anion-exchange resin available in the chloride form (sevelamer hydrochloride) and in the buffered form with bicarbonate (sevelamer carbonate). The safety and efficacy of these two compounds are equivalent, although some patients treated with sevelamer hydrochloride may develop more gastrointestinal side effects, such as vomiting and diarrhea, and metabolic acidosis due to the intestinal exchange of chloride for bicarbonate [85]. Treatment with sevelamer is additionally accompanied with an overall anti-inflammatory effect, lowering of LDL-cholesterol, and uric acid levels [86,87]. Compared to calcium-based compounds, sevelamer treatment resulted in lower incidence of hypercalcemia, decreased incidence of low PTH, and improvement of bone formation rate and trabecular architecture [88]. Moreover, sevelamer has also shown to reduce circulating FGF-23 levels, and thus potentially reduces the risk of left ventricular hypertrophy [89].

Lanthanum carbonate contains the trivalent cation lanthanum, and it forms a water-insoluble compound, lanthanum phosphate, in the gut [90]. According to an in vivo study with rats, lanthanum carbonate appeared to be more effective than calcium carbonate or sevelamer in reducing urine phosphate excretion, and in increasing excretion in feces, this being indicative of phosphate binding potency [91]. In longer-term studies, treatment with lanthanum carbonate appeared to be as effective as conventional phosphate binder therapy, including calcium carbonate, in improving hyperphosphatemia [92,93]. The most common adverse events were of a gastrointestinal nature (such as nausea, vomiting, diarrhea, and constipation) and of mild-to-moderate severity. Accumulation of lanthanum carbonate in blood and bone was below toxic levels [94]. In a 1-year phase III clinical trial including 98 patients, the effect of lanthanum carbonate was compared with calcium carbonate on the evolution of renal bone disease in dialysis patients [92]. Incidence of hypercalcemia was significantly lower in the lanthanum-treated group compared with the calcium-treated group (6% vs. 49%, respectively). In the lanthanum carbonate group, fewer recipients had renal osteodistrophy at end-of-study than at baseline, and most of those with baseline low bone turnover and baseline hyperparathyroidism evolved toward a normalization of their bone turnover, whereas in the calcium carbonate group, numerically more patients had renal bone disease at study end than at baseline. According to the LANDMARK (lanthanum carbonate compared with calcium carbonate in hemodialysis patients) trial, a randomized clinical study involving 2374 patients with CKD undergoing hemodialysis, treatment of hyperphosphatemia with lanthanum carbonate in comparison with calcium carbonate did not result in any difference in terms of composite cardiovascular events (hazard ratio 1.11, 95% CI 0.88–1.41, *p*-value 0.37) [95]. 

The new generation of calcium-free and aluminum-free phosphate-lowering agents is represented by iron-based phosphate binders. Ferric citrate and sucroferric oxyhydroxide are the two iron-based phosphate binders clinically approved for safety and efficacy for the treatment of hyperphosphatemia [11]. The use of ferric citrate provides the added benefit of improved iron parameters, including increases in ferritin, iron, and transferrin saturation (TSAT) [96] that may result in a significant reduction in the need for erythropoiesis-stimulating agents (ESA) and intravenous iron therapy, with a consequent reduction in costs [97]. In turn, ferric citrate is associated with the risk of clinically evident iron overload. On the contrary, sucroferric oxyhydroxide is associated with a low risk of iron accumulation because of minimal systemic iron absorption [98]. Moreover, it can achieve similar efficacy to sevelamer carbonate with a lower daily pill burden compared to sevelamer, which may guarantee better treatment adherence in clinical practice [99]. The most frequent side effects observed for both sucroferric oxyhydroxide and ferric citrate are gastrointestinal related, including discolored black feces, mild-to-moderate transient diarrhea, abdominal distension, and constipation.

#### 4.1.3. Phosphate Removal through Dialysis for Patients with CKD Stage G5D

Removal of phosphate through dialysis in CKD patient stage G5 is dependent on the type of dialysis, session length, and dialysate flow. For patients who perform peritoneal dialysis four times per day with 2 L exchanges, an estimated amount of 2.0–2.2 g of phosphate is removed per week. In patients on conventional three-times-a-week, 4 h per session hemodialysis schedule, the amount of phosphate removed is approximately 2.3–2.6 g, only half of the assumed dietary intake, if limited to 800 mg/day [100]. Thence, to achieve the goal of neutral phosphate balance, the administration of phosphate-lowering agents is mandatory to control serum phosphate concentration in dialysis patients.

### 4.2. Treatment of Secondary Hyperparathyroidism

As kidney function declines, incidence and severity of SHPT increases and leads to abnormalities in bone turnover and mineralization. For patients with CKD stage G5 with SHPT, the updated 2017 guidelines do not clarify which is the first-line therapy to administer but it recommends the use of calcimimetics, calcitriol, or vitamin D analogues in monotherapy, or a combination of calcimimetics with calcitriol or vitamin D analogues [71]. Moreover, in case of severe hyperparathyroidism unresponsive to pharmacological therapy, parathyroidectomy is suggested [69].

#### 4.2.1. Vitamin D and Analogues

Vitamin D is a group of fat-soluble secosteroids involved in the regulation of gene transcription in target tissues through the binding to its nuclear vitamin D receptor (VDR). Both the Kidney Disease Outcomes Quality Initiative (KDOQI) and Kidney Disease Improving Global Outcomes (KDIGO) guidelines recommend the correction of hypovitaminosis D through nutritional vitamin D replacement in CKD and dialysis patients to prevent SHPT [71,72]. The recommended target level of vitamin D is >30 ng/mL, whereas vitamin D deficiency is defined as serum vitamin D level < 20 ng/mL and vitamin D insufficiency as between 20 and 29 ng/mL [101].

The major natural source of vitamin D is acquired from sunlight-induced cutaneous synthesis, and the rest derives from diet as ergocalciferol (D2) from vegetable sources and cholecalciferol (D3) from animal sources. Many vitamin D supplementation regimens use either ergocalciferol or cholecalciferol on a daily, weekly, or monthly basis. Vitamin D precursors are hydroxylated in the liver to calcifediol (25-hydroxycholecalciferol, 25[OH]D), the major circulating form of vitamin D. Calcifediol is therefore hydroxylated in the kidneys to calcitriol (1,25-dihydroxycholecalciferol), the active form of vitamin D, which activates the vitamin D receptor in the cellular nucleus. Calcitriol deficiency results in a decreased intestinal absorption of calcium and may lead to hypocalcemia, which further stimulates PTH secretion. Calcitriol is available as capsules, an oral solution, and an intravenous solution. Synthetic activated vitamin D analogues, also known as vitamin D receptor activators (VDRAs), include paricalcitol, doxercalciferol, alfacalcidol, and others. Multiple RCTs reported significant reductions of PTH levels with calcitriol or vitamin analogues compared to placebo [102,103,104,105]. However, two RCTs, PRIMO (paricalcitol capsule benefits in renal failure-induced cardiac morbidity) and OPERA (oral paricalcitol in stage 3–5 chronic kidney disease) trials, demonstrated a significant increased risk of hypercalcemia in patients with CKD stages 3–5 treated with paricalcitol in the absence of clinically beneficial effects on cardiac endpoints, and in particular it did not alter left ventricular mass index or improve its function [104,105]. On the basis of these results, KDIGO Work Group no longer recommends the routine use of calcitriol or analogues in non-dialysis CKD (ND-CKD) patients with moderate SHPT, but that they should be reserved for only severe and progressive SHPT [71]. A secondary analysis of the cross-sectional VIKI (vitamin K Italian) study showed a significant association between oral calcitriol and reduction in vertebral fracture risk in patients undergoing hemodialysis without an increase in the burden of vascular calcifications [106]. In the phase 4 randomized IMPACT-SHPT study (study to evaluate the improved management of iPTH with paricalcitol-centered therapy vs. cinacalcet therapy with low-dose vitamin D in hemodialysis patients with secondary hyperparathyroidism) on 272 patients with CKD and SHPT on dialysis, paricalcitol significantly reduced bone turnover markers and PTH levels [107], but increased FGF-23, calcium, and phosphorus levels, compared to cinacalcet [108]. Similarly, in the randomized PARADIGM study (a randomized trial of cinacalcet versus vitamin D analogs as monotherapy in Secondary hyperparathyroididm) in 312 patients on dialysis, treatment of SHPT with vitamin D analogue was associated with increased FGF-23 concentration, whereas cinacalcet use was associated with a decrease in FGF-23 [109,110]. Both trials showed that paricalcitol and cinacalcet had comparable effects on the control of PTH concentrations. Furthermore, paricalcitol seems to have a significant antiproteinuric effect both in ND-CKD [111] and kidney transplant recipients [112] as a result of intrarenal direct inhibition of the transcription of the gene encoding for renin. 

#### 4.2.2. Calcimimetic Agents

Cinacalcet hydrochloride is the first calcimimetic drug approved for the treatment of SHPT in adult dialysis patients. It regulates the synthesis and secretion of PTH by increasing the sensitivity of the calcium-sensing receptor (CaSR) to extracellular calcium ions [113]. It is taken orally, and it provides a rapid, dose-dependent reduction in plasma PTH levels that persists for up to 24 h after drug administration [114]. In two large phase 3, randomized, double-blind, multicentre, clinical trials involving in total 741 patients undergoing hemodialysis, a significantly higher proportion of patients receiving cinacalcet 30–180 mg/day achieved a reduction in PTH levels to < or = 250 pg/mL, compared to placebo [115]. In addition, cinacalcet lowers serum calcium and phosphorus, calcium-phosphorous product levels, and it reduces high bone turnover [116]. However, in the large, randomized placebo-controlled, double-blind EVOLVE (effect of cinacalcet on cardiovascular disease in patients undergoing dialysis) trial conducted in 3883 dialysis patients with SHPT, cinacalcet failed to meet the primary composite endpoint, defined as reduction in the risk of death or hospitalization due to major cardiovascular events or from any cause [117]. Next, a secondary analysis of EVOLVE trial showed that cinacalcet decreased the risk of death and of major cardiovascular events in older, but not in younger patients, suggesting that this treatment should be tailored on the basis of the individual risk profile; cardiovascular effect modification by age may be partly explained by differences in baseline cardiovascular risk (older patients are more susceptible to the beneficial effect of cinacalcet) and by differential application of cointerventions that reduce PTH (including kidney transplantation, parathyroidectomy, and commercial cinacalcet use) [118]. Moreover, it has been demonstrated a significant risk reduction for severe unremitting HPT requiring parathyroidectomy [119] or the development of calciphylaxis with the use of cinacalcet [120]. Treatment with cinacalcet is generally well tolerated. The most frequent side effects are gastrointestinal, primarily nausea and vomiting. Episodes of hypocalcemia are mostly mild and asymptomatic. In the cinacalcet arm of the EVOLVE trial, no negative events were associated with the persistently low serum calcium levels [117].

Etelcalcetide is a second-generation calcimimetic that has been approved for the treatment of SHPT in adult hemodialysis patients. It interacts with the CaSR at a site distinct from cinacalcet [121]. Etelcalcetide has proven to be more effective in lowering PTH when compared to cinacalcet, as demonstrated by the double-blind RCT conducted by Block et al. in 683 dialysis patients with serum PTH concentrations > 500 pg/mL [122]. The use of etelcalcetide was not inferior to cinacalcet in reducing PTH concentrations (primary end point), but it appeared to be superior on secondary endpoint; in fact, 52.4% of patients randomized to etelcalcetide vs. 40.2% of patients randomized to cinacalcet experienced a 50% reduction in PTH concentrations from baseline. In addition, etelcalcetide allowed a more potent reduction in serum concentrations of FGF-23 and two markers of high-turnover bone disease (bone-specific alkaline phosphate and collagen type I cross-linked C) [122]. Tolerability and safety were similar between the two groups. There were no differences in self-reported nausea and vomiting. On the pharmacokinetic point of view, etelcalcetide is almost exclusively cleared by the kidney through glomerular filtration; therefore, its half-life significantly increases with declining renal function, determining sustained reductions in PTH for up to 72 h in patients on hemodialysis [123]. The longer half-life allows intravenous administration thrice weekly concurrent with a hemodialysis session, with consequent better drug adherence and reduction in pill burden. As with cinacalcet, the use of etelcalcetide may be accompanied by reduced serum calcium concentrations and asymptomatic hypocalcemia that in the most of patients does not require modification of SHPT treatment. Notably, hypocalcemia is more common with etelcalcetide than with cinacalcet [122].

#### 4.2.3. Parathyroidectomy

According to 2017 KDIGO guidelines, the proposed PTH target is suggested to be maintained in the range of approximately two to nine times the upper normal limit (130–600 pg/mL) [71]. The early stage of SHPT is characterized by a diffuse hyperplasia with polyclonal parathyroid cell proliferation; in this phase, pharmacological therapy (i.e., phosphorus-lowering agents, vitamin D analogs, and calcimimetics) effectively reduces high serum PTH concentration. If SHPT is not treated appropriately, a nodular hyperplasia with adenomatous tissue can develop and become resistant to therapies because of lowered expression of CaSR and VDR [40]. In patients with CKD from G3a to G5D with severe SHPT that is refractory to pharmacological therapy, parathyroidectomy is indicated [71]. Surgical options are total and subtotal parathyroidectomy. Total parathyroidectomy consists in total removal of all identifiable parathyroid tissue, leading to postoperative complications, such as adynamic bone disease and severe hypocalcemia. In order to support bone mineral homeostasis, autotransplantation after total prathyroidectomy allows to preserve a residual parathyroid tissue—a fragment of gland is transplanted into the forearm or sternocleidomastoid muscle or subcutaneous adipose tissue. Subtotal parathyroidectomy consists in the removal of three and a half parathyroid glands, with the preservation of a gland fragment in its original site within the neck and reduced risk of postoperative hypocalcemia [3]. A meta-analysis of 13 studies including 1589 patients undergoing dialysis showed that both subtotal and total parathyroidectomy were effective and safe in treating SHPT and that differences in recurrence of HPT and reintervention rate were not statistically significant across treatment groups [124]. 

### 4.3. Treatment of CKD–MBD/Osteoporosis

Bisphosphonates are currently the treatment of choice for osteoporosis in the general population. They are a group of chemically stable derivatives of inorganic pyrophosphate with a very high affinity for bone mineral [125] and include risedronate, alendronate, pamidronate, ibandronate, and zoledronic acid. By binding to hydroxyapatite crystals in sites of active bone remodeling, bisphosphonates inhibit hydroxyapatite breakdown and osteoclast bone resorption, and promote osteoclast apoptosis [125]. The share of bisphosphonates not retained in the bone is cleared from circulation by renal excretion. In patients with osteoporosis and CKD without evidence of CKD–MBD, the use of bisphosphonates should follow general population guidelines [69,126]. However, since bisphosphonates have a long half-life and could potentially accumulate in the bone of patients with compromised renal function, they are not recommended in patients with CKD stage from 4 to 5. Adverse effects of bisphosphonates therapy include osteonecrosis of the jaw [127], atypical femoral fracture [128], and severe hypocalcemia [129].

Denosumab is a fully human monoclonal antibody that binds the receptor activator of nuclear factor-κB ligand (RANKL) with high affinity and specificity and it prevents the interaction of RANKL with its receptor RANK on osteoclasts and their precursors [130]. By binding RANKL, denosumab inhibits formation and activity of osteoclasts, decreases bone resorption, and increases bone mineral density [131]. In the randomized, placebo-controlled FREEDOM trial (fracture reduction evaluation of denosumab in osteoporosis every 6 months) in postmenopausal women with osteoporosis, treatment with denosumab over 36 months was associated with a reduction in the risk of vertebral, nonvertebral, and hip fractures [132]. Observations from the FREEDOM study extension showed that denosumab treatment through 5 years resulted in normal bone quality with reduced bone resorption [133]. A secondary data analysis conducted among patients with CKD participating in the FREEDOM trial showed that the efficacy and safety of denosumab does not depend on kidney function [134]. Similarly, in an open-label, single-dose, multicenter study in subjects with various degrees of renal function impairment, it was demonstrated that denosumab pharmacokinetics and pharmacodynamic were not modified by renal function, suggesting denosumab dose adjustment based on glomerular filtration rate is not required [135]. The only treatment-related serious adverse event reported during denosumab therapy was hypocalcemia. Risk factors for hypocalcemia include lower baseline serum calcium and 25(OH)vitamin D. As a precaution, it is generally recommended to administer supplementation of calcium and active vitamin D when patients with reduced renal function initiate denosumab therapy to prevent hypocalcemia [135,136]. Serum calcium levels usually reach the lowest point around seven days after administration of denosumab, with a less extensive serum calcium decrease with the second administration [137].

**Table 2:** Randomized clinical trials about different treatments acting on CKD–MBD. CaMg calcium acetate/magnesium carbonate, CKD chronic kidney disease, CV cardiovascular, HD hemodialysis, LC lanthanum carbonate, LV left ventricular, and PTH parathormone.

Study (Year)	Type	Drugs	Sample Size and Population	Outcome	Results
CALMAG De Francisco (2010) [82]	Phase 4	Calcium acetate/magnesium carbonate vs. sevelamer hydrochloride	326 patients HD	Efficacy of CaMg compared with sevelamer-HCl as an active control of serum phosphorus at week 25.	CaMg was noninferior to the comparator at controlling serum phosphorus levels at week 25.
D’Haese (2003) [92]	Phase 3	Lanthanum carbonate vs. calcium carbonate	98 Patients HD	Tolerability, phosphate binder efficacy, incidence of hypercalcemia, and evolution to low bone turnover	LC-treated patients show almost no evolution toward low bone turnover over one year.
LANDMARK Ogata (2021) [95]	Phase 3	Lanthanum carbonate vs. Calcium carbonate	2374 patients HD	Reduction in cardiovascular events. Overall survival, secondary hyperparathyroidism-free survival, hip fracture-free survival, and adverse events.	Treatment of hyperphosphatemia with LC compared with CC did not result in a significant difference in composite CV events.
PRIMO Thadhani (2012) [104]	Phase 3	Paricalcitol vs. placebo	227 CKD stages 3–4 patients with LV hypertrophy, preserved left ventricular ejection fraction	Change in LV mass index over 48 weeks by cardiac magnetic resonance imaging. Echocardiographic changes in left ventricular diastolic function.	Paricalcitol did not alter left ventricular mass index or improve diastolic dysfunction.
OPERA Wang (2014) [105]	Not applicable	Paricalcitol vs. placebo	60 CKD stages 3–5 patients with LV hypertrophy	Change in LV mass index over 52 weeks by cardiac magnetic resonance imaging. Changes in LV volume, echocardiographic measures of systolic and diastolic function, biochemical parameters of MBD, and measures of renal function.	52 weeks of treatment with oral paricalcitol significantly improved secondary hyperparathyroidism but did not alter measures of LV structure and function.
IMPACT-SHPT Ketteler (2012) [107]	Phase 4	Paricalcitol vs. cinacalcet	272 patients HD	PTH 150–300 pg/mL	Overall superiority of paricalcitol (56.0%) over cinacalcet (38.2%; *p* = 0.010) in achieving PTH 150–300 pg/mL during Weeks 21–28.
PARADIGM Wetmore (2015) [110]	Phase 4	Cinacalcet vs. vitamin D analogs	312 patients HD	Mean percentage change in plasma PTH levels. Proportion of participants achieving plasma PTH <300 pg/mL or a ≥30% decrease in PTH.	Modest reductions in PTH with either cinacalcet or vitamin D analog monotherapy over 52 weeks of treatment.
EVOLVE (2012) [117]	Phase 3	Cinacalcet vs. placebo	3883 patients HD	All-cause mortality, major cardiovascular events, development of severe unremitting HPT.	Cinacalcet did not significantly reduce the risk of death or major cardiovascular events
Block (2017) [122]	Phase 3	Etelcalcetide vs. cinacalcet	683 patients HD	Noninferiority of etelcalcetide at achieving more than a 30% reduction from baseline in PHT compared to cinacalcet. Superiority in achieving >50% and >30% reduction in PTH. Self-reported nausea and vomiting.	Non-inferiority of etelcalcetide in reduction in PTH concentrations compared to cinacalcet.
FREEDOM Cummings (2009) [132]	Phase 3	Denosumab vs. placebo	7868 postmenopausal women with osteoporosis	New vertebral fractures. Nonvertebral and hip fractures.	Denosumab given subcutaneously twice yearly for 36 months was associated with a reduction in the risk of vertebral, nonvertebral, and hip fractures in women with osteoporosis.

## 5. Conclusions

CKD–MBD is a very complex and multifactorial disease. Despite the big effort to improve the management of patients with CKD–MBD, several unmet needs still remain. In particular, the true cut-off of risk factors such as serum phosphate, PTH, FGF23, and the magnitude of association of novel drugs targeting MBD pathways and future CV, renal and mortality risk deserve further studies in the future. 

## Figures and Tables

**Table 1:** Observational studies in MBD. CKD chronic kidney disease, CV cardiovascular, eGFR estimated glomerular filtration, ESKD end stage kidney disease, FgF23 fibroblast growth factor 23, HD hemodialysis, PD peritoneal dialysis, and PTH parathormone.

Study (Year)	Type	Population	Sample Size	Outcome	Results
Lopes et al. (2020) [56]	Prospective cohort study	HD	17,414 patients	CV mortality	Patients with poor control of serum phosphorus levels (expressed as AUC) during a 6-month period have a higher risk of CV mortality (for AUC > 2 = HR 2.03, 95% CI 1.53–2.69).
Hou Y. et al. (2017) [57]	Meta-analysis of 9 cohort studies	HD or PD	1,992,869 patients	All-cause mortality	Compared to reference phosphorus category, both very high (HR 1.39; 95% CI 1.31–1.47) and very low (HR 1.16, 95% CI 1.06–1.28) phosphorus levels are associated with a greater risk for all-cause mortality.
Dhingra et al. (2007) [58]	Observational prospective study	Not CKD	3368 patients	Incident of Cardiovascular disease	Patients with elevated phosphorus serum levels have a greater risk of CVD (HR 1.55, 95% CI 1.16–2.07%; *p* = 0.004).
Bellasi et al. (2011) [59]	Observational retrospective study	CKD 3–5, not requiring dialysis	1716 patients	Composite end point of progression to ESKD or death	A worse control of phosphorus levels (≥4.3 mg/dL) exposes to an increased risk of progression to ESKD or death (HR ratio 2.04; 95% CI 1.44–2.90).
Johhn J. Sim et al. (2013) [60]	Observational retrospective longitudinal study	Not CKD	94,989 patients	Incident of ESKD	Iperphosphatemia is associated with greater risk for ESKD. Risk increased by 40% for each increase of 0.5 mg/dL of serum phosphate.
Yang et al. (2016) [65]	Meta-analysis of 7 studies	HD or PD	1406 patients	All-cause mortality	Higher serum FGF23 levels are associated with increased risk of death (HR 1.53; 95% CI: 1.05–2.25).
Rebholz et al. (2015) [67]	Observational prospective study	Not CKD	13,488 patients	Incident of ESKD	Elevated fibroblast growth factor serum concentration is associated with greater risk of ESKD (HR 2.10; 95% CI 1.31 to 3.36; trend *p* < 0.001).
Isakova et al. (2011) [12]	Observational prospective study	CKD 2–4	3879 patients	All-cause mortality and ESKD	Patients with CKD 2–4 with higher FGF23 levels have an augmented risk of death (quartile 1, reference; quartile 2, HR 1.3; 95%CI 0.8–2.2; quartile 3, HR 2.0; 95%CI 1.2–3.3; quartile 4, and HR 3.0; 95%CI 1.8–5.1). Elevated FGF23 values are associated with higher risk of ESKD (for patients with eGFR 30–44 mL/min = HR 1.3 per SD of lnFGF23; 95%CI 1.04–1.6 and for patients with eGFR ≥ 45 mL/min = HR 1.7; 95% CI 1.1–2.4). This association has not been demonstrated for patients with eGFR< 30 mL/min.
Borrelli et al. (2018) [68]	Observational prospective study	CKD 1–5, not requiring dialysis	543 patients	Renal death (composite of ESKD or all-causes death before ESKD)	In the same subject, higher PTH variation over time (∆PTH) with respect to baseline value is associated with an augmented risk of renal death (for the highest ΔPTH quartile = HR 1.91; 95%CI:1.08–3.38; *p* = 0.026).
Floege J. et al. (2011) [70]	Observational prospective study	HD	7970 patients	All-cause mortality	Compared to reference PTH range (150–300 pg/mL), both elevated (>600 pg/mL) and low (<75 pg/mL) PTH levels are, respectively, associated with a 2-fold (HR 2.10; 95%CI 1.62–2.73, *p* < 0.001) and 1,5-fold (HR 1.46; 95% CI 1.17–1.83, *p* = 0.001) risk of death.

## Data Availability

Not applicable.
